# Supplementary material for: The relationship between reinforcement and explicit control during visuomotor adaptation
Source: Sci Rep. 2018 Jun 14;8:9121. doi: 10.1038/s41598-018-27378-1 (PMC6002524; doi:10.1038/s41598-018-27378-1)
Supplement: Supplementary file 1 — Supplementary figures [file 41598_2018_27378_MOESM1_ESM.docx]

*Supplementary figures for:*

***The relationship between reinforcement and explicit control during visuomotor adaptation***

**Olivier Codol**^1*^**, Peter J Holland**^1^**& Joseph M Galea**^1^

^1^School of Psychology, University of Birmingham, UK

* Corresponding author

Correspondence:

Olivier Codol

School of Psychology

University of Birmingham, UK

Email: codol.olivier@gmail.com


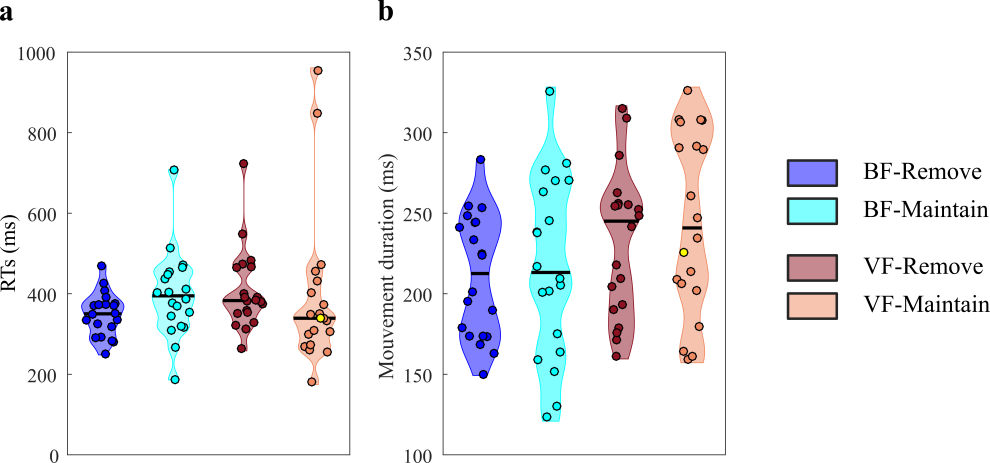


**Supplementary figure S1. Experiment 1: feedback-instruction. (a)** Average reaction times of participants during the asymptote phase. **(b)** Average movement duration of participants during the asymptote phase. Each dot represents one participant. The yellow dot represents the same participant across all plots (the same participant as figure 2). Black lines are group medians and the shaded areas indicate distribution of individual values.


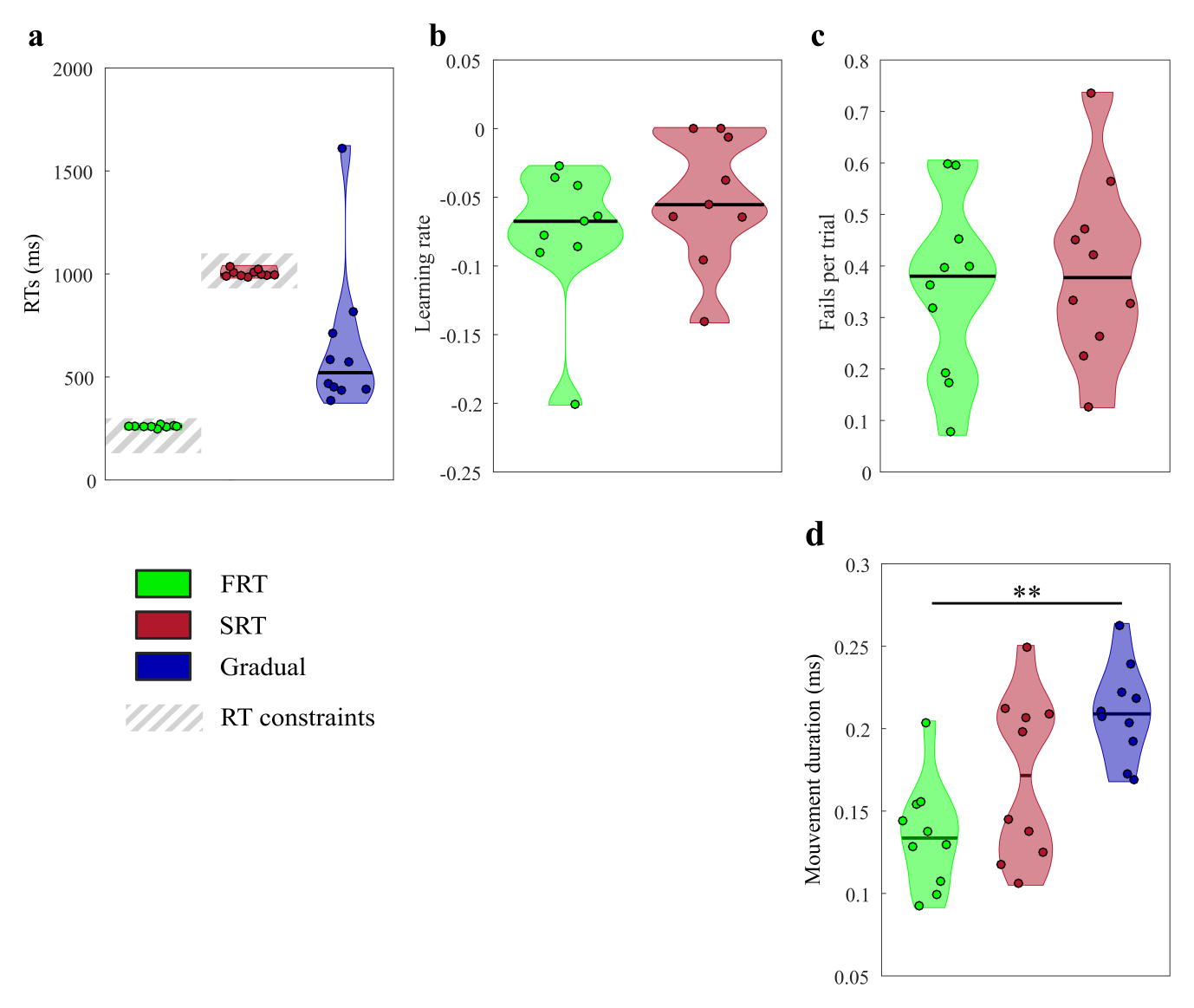


**Supplementary figure S2. Experiment 2: forced RT. (a)** Average reaction times of participants throughout the task. **(b)** Average number of failures per trial to initiate movements within the constrained timeframe throughout the task. **(c)** Average movement duration of participants throughout the task. **(d)** Learning rates during the adaptation phase. Each dot represents one participant. Black lines are group medians and the shaded areas indicate distribution of individual values. SRT: short reaction time; FRT: fast reaction time. ** p<0.01.


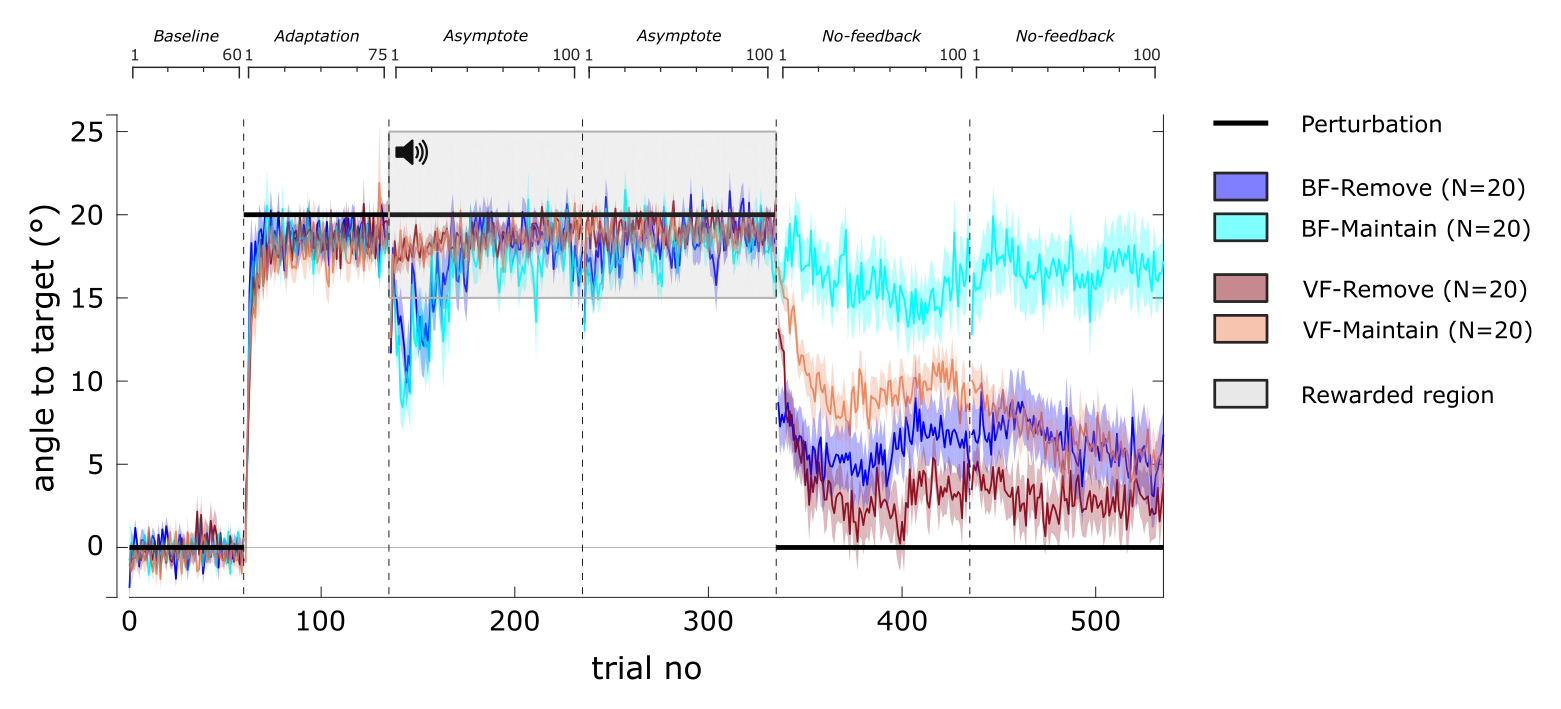


**Supplementary figure S3. Trial-by-trial reach angles for experiment 1.** Reach angles with respect to target (˚) of each group during the visuomotor displacement task. Vertical bars represent block limits. The binary feedback consisted of a pleasant sound in the rewarded region. The black solid line represents the hand-to-cursor discrepancy (the perturbation) for all groups across the task. The upper and lower horizontal axes represent block-relative and absolute trial number, respectively. Coloured lines represent group mean and shaded areas represent s.e.m.
